# Genomic detection of Panton-Valentine Leucocidins encoding genes, virulence factors and distribution of antiseptic resistance determinants among Methicillin-resistant *S. aureus* isolates from patients attending regional referral hospitals in Tanzania

**DOI:** 10.1186/s12920-025-02085-9

**Published:** 2025-01-20

**Authors:** Masoud A. Juma, Tolbert Sonda, Boaz Wadugu, Davis Kuchaka, Mariana Shayo, Petro Paulo, Patrick Kimu, Livin E. Kanje, Melkiory Beti, Marco Van Zwetselaar, Blandina Mmbaga, Happiness Kumburu

**Affiliations:** 1 Kilimanjaro Christian Medical University College, Kilimanjaro, Tanzania; 2 Kilimanjaro Clinical Research Institute, Kilimanjaro, Tanzania; 3 Department of Microbiology, State University of Zanzibar, Zanzibar, Tanzania; 4 Kilimanjaro Christian Medical Centre, Kilimanjaro, Tanzania

**Keywords:** Panton valentine leucocidin (pvl), Quaternary ammonium compounds (*Qac*), Sequence type (ST), Methicillin-resistant *S. aureus* (MRSA)

## Abstract

**Background:**

Methicillin-resistant *Staphylococcus aureus* (MRSA) is a formidable public scourge causing worldwide mild to severe life-threatening infections. The ability of this strain to swiftly spread, evolve, and acquire resistance genes and virulence factors such as pvl genes has further rendered this strain difficult to treat. Of concern, is a recently recognized ability to resist antiseptic/disinfectant agents used as an essential part of treatment and infection control practices. This study aimed at detecting the presence of pvl genes and determining the distribution of antiseptic resistance genes in Methicillin-resistant *Staphylococcus aureus* isolates through whole genome sequencing technology.

**Materials and methods:**

A descriptive cross-sectional study was conducted across six regional referral hospitals-Dodoma, Songea, Kitete-Kigoma, Morogoro, and Tabora on the mainland, and Mnazi Mmoja from Zanzibar islands counterparts using the archived isolates of *Staphylococcus aureus* bacteria. The isolates were collected from Inpatients and Outpatients who attended these hospitals from January 2020 to Dec 2021. Bacterial analysis was carried out using classical microbiological techniques and whole genome sequencing (WGS) using the Illumina Nextseq 550 sequencer platform. Several bioinformatic tools were used, KmerFinder 3.2 was used for species identification, MLST 2.0 tool was used for Multilocus Sequence Typing and SCCmecFinder 1.2 was used for SCC*mec* typing. Virulence genes were detected using virulenceFinder 2.0, while resistance genes were detected by ResFinder 4.1, and phylogenetic relatedness was determined by CSI Phylogeny 1.4 tools.

**Results:**

Out of the 80 MRSA isolates analyzed, 11 (14%) were found to harbor *LukS-PV* and *LukF-PV*, pvl-encoding genes in their genome; therefore *pvl*-positive MRSA. The majority (82%) of the MRSA isolates bearing pvl genes were also found to exhibit the antiseptic/disinfectant genes in their genome. Moreover, all (80) sequenced MRSA isolates were found to harbor SCC*mec* type IV subtype 2B&5. The isolates exhibited 4 different sequence types, ST8, ST88, ST789 and ST121. Notably, the predominant sequence type among the isolates was ST8 72 (90%).

**Conclusion:**

The notably high rate of antiseptic resistance particularly in the Methicillin-resistant *S. aureus* strains poses a significant challenge to infection control measures. The fact that some of these virulent strains harbor the *LukS-PV* and *LukF-PV*, the pvl encoding genes, highlight the importance of developing effective interventions to combat the spreading of these pathogenic bacterial strains. Certainly, strengthening antimicrobial resistance surveillance and stewardship will ultimately reduce the selection pressure, improve the patient’s treatment outcome and public health in Tanzania.

## Introduction

The escalating threat of antibiotic resistance has been well known to impose a serious burden on public health, particularly in the treatment and control of infectious diseases [1]. Methicillin-resistant *S. aureus* (MRSA) has become a formidable resistant strain whose resistance extended not only to β-lactams antibiotics but to other non-β-lactams and even further to antiseptic and disinfectant agents [2, 3]. The latter has limited the effect of disinfectant agents which have been long used in infection control practices and are critical components in the prevention of nosocomially spreading of infections [4]. This antiseptic resistance is mediated by different resistant determinants such as small multidrug-resistant genes (*smr*) but *qac* genes are worth mentioning [4–6]. These *qac* genes mediate resistance to DNA-intercalating agents (ethidium bromide) and quaternary ammonium compounds such as benzalkonium chloride and chlorhexidine, which are the widely used disinfectants [6, 7]. Several *qac* genes are known to exist such as *qacA*, *qacB*,* qacC*, and *qacD* [5]. It is worth noting that, these aforementioned genes are plasmid-borne and mediate the resistance through encoding the multiprotein channels that catalyze the efflux of disinfectant agents [8]. Nevertheless, the fact that these antiseptic resistant genes [*qac*) are found in mobile genetic elements, this renders them susceptible to be horizontally transmitted among various species of staphylococcus and consequently, widening the problem of disinfectant resistance [4, 9].

Moreover, the secretion of toxins is another characteristic that enhances the severity of *S. aureus* diseases, these include enterotoxin, exfoliating toxin, and toxic shock syndrome toxin [10, 11]. However, an even more potent and aggressive toxin is a pore-forming toxin called Panton valentine Leukocidin toxin which kills neutrophils outright through the introduction of pores into their plasma membranes [12, 13]. This triggers extensive inflammation, inflicts severe tissue damage, and ultimately increases the severity of infections [14]. Indeed, the Panton Valentine Leucocidins toxin is encoded by two co-transcribed phage-borne genes called *LukS-PV* and *LukF-PV*, whose expression results in eight protein subunits of LukS and LukF that reassemble to form pores disrupting the cell membranes of host neutrophils [15, 16]. It is noteworthy that, the MRSA strains bearing the Pvl- encoding genes tend to be more virulent causing more severe skin and soft tissue infections as well as other life-threatening invasive infections [13, 17]. Consequently, the presence of pvl-encoding gene has not only increased their pathogenicity but rendered them more transmissible as well [13, 14, 18].

In Tanzania, like many other resource-limited countries in Africa, studies on detailed genotypic characteristics and resistance profiles of MRSA are limited even amidst the extensive use of disinfectants/antiseptics in our hospitals. This has consequently created a knowledge gap and diminishes the correct preventive and control measures against this multidrug resistant strain, which is persistent and spread swiftly. Notably, many studies in Tanzania have targeted bacterial antibiotic resistance with none or few studies detailing their antiseptic resistance and virulence genes. Therefore, this study aimed at performing extended analysis at the genetic level to provide useful molecular information on resistance profile, virulence genes, sequence typing, and phylogenetic relationship of MRSA isolates using whole genome sequencing technique.

## Materials and methods

### Study design and sites

A descriptive cross-sectional laboratory-based study was conducted using the archived isolates of *S. aureus* bacteria collected from different patients who attended six Tanzanian regional referral hospitals from January 2020 to December 2021. These isolates were collected during a parent study of SeqAfrica, aimed to combat antimicrobial resistance and capacity building in Africa region. In Tanzania, this study involved several regional referral hospitals. These hospitals are Mnazi Mmoja Referral Hospital of Zanzibar, Tabora, Dodoma, Songea, Kigoma, and Morogoro Regional Referral Hospitals. The archived isolates of *S. aureus* identified as Methicillin-resistant *S. aureus* collected within the specified timeframe with all necessary demographic and culture result data were included in this study. The MRSA characteristic was identified by conventional methods and confirmed by molecular methods through the presence of the *mecA* gene.

### Subculturing, DNA extraction, library preparation, and sequencing

The isolates were retrieved from − 80^o^C, allowed to attain room temperature before being subcultured in Blood Agar media, and incubated overnight at 35^o^C. Species identification was done using both phenotypic and genotypic approaches. Phenotypic re-identification was respectively performed using gram stain, catalase, and coagulase tests using inhouse standard operating procedures. Genotypic identification was performed by whole genome sequencing method. Prior to genotypic identification, DNA extraction was done using Quick-DNA™ Fungal/Bacteria Miniprep Kit per the manufacturer’s instructions. a Qubit^®^ version 4.0 fluorometer was then used for quantification of the extracted genomic DNA samples. Library preparation was performed based on the NEBNext^®^ Ultra™ II FS DNA Library Prep Kit manual and Illumina Nextseq550 next-generation sequencer (2 × 250 bp paired-end reads protocol). Briefly, library preparation involves fragmentation of DNA, adaptor ligation, size selection, barcoding, and pooling of each extracted genomic DNA. After preparation, the library was normalized and mixed with a PhiX control, then loaded onto the Illumina Nextseq 550 sequencer for DNA sequencing.

The isolates were retrieved from a -80^o^C deep freezer, allowed to gain room temperature, and then subcultured on Blood Agar media. Phenotypic re-identification of *Staphylococcus aureus* was performed using Gram staining, catalase testing, and confirmed by coagulase tests. Quality control was maintained using *S. aureus* ATCC 29,313 and ATCC 25,923 strains as reference organisms. All procedures followed standard operating protocols established by the Microbiology Laboratory Department at the KCRI Biotechnology Laboratory.

### Statistical analysis

SPSS version 20 was used to analyze and summarize the demographic and culture results data using frequency and proportions.

### Bioinformatics

Trimming of adapters, filtering of the short reads and quality control check of the raw reads were done using a Trimmomatic tool 0.39 and FastQC 0.12.0. Importantly, De novo assembly was performed using St. Petersburg genome assembler (SPades) 3.15.5 tool, whereas the Fasta files format containing contigs were obtained. The sequence data were then further analyzed using CGE tools found in a Bacterial Analysis Pipeline (BAP 3.3.2), online available at www.genomicepidemiology.org. The species identification of the sequenced isolates was determined using KmerFinder 3.2 [19]. Multilocus Sequence typing based on sequencing seven housekeeping genes (*pta*, *arcC*, *tpi*, *aroE*, *gmk*, *yqiL*, and *glpF*), and Sequence types (STs) were determined using MLST 2.0 [20]. Furthermore, virulence genes, and SCC*mec* type were also determined by using virulenceFinder 2.0, and SCC*mec* Finder 1.2 respectively [21–24]. For analysis of genetic relatedness, single nucleotide polymorphism (SNPs) was analyzed using CSI Phylogeny 1.4 was used [25]. The tool generated Newick files which was used for further annotation and visualization of the phylogenetic tree by using Figtree 1.4.4 software [20]. The raw reads and genome assemblies of all eighty isolates from this study have been deposited in the European Nucleotide Archive (http://www.ebi.ac.uk/ena) under accession number PRJEB71932.

## Results

### Clinical and demographic details of the study isolates

A total of 80 isolates confirmed as Methicillin-resistant *S. aureus* bacteria by both phenotypic and genotypic methods were included in this study. Morogoro, Dodoma, Mnazi Mmoja, and Kitete Referral hospitals contributed about 37 (46.3%), 18 (22.5%), 14 (17.5%), and 10 (12.5%) number of the isolates, respectively. The other Referral Hospital, namely, Maweni Regional Referral Hospital of Kigoma contributed 1 (1.3%) isolate only. The majority 46 (57.5%) of the isolates were from the wound pus, while 17(21.3%), and 14 (17.5%) recovered from urine and blood respectively. It is noteworthy, that most of these bacteria, 51 (63.8%) were obtained from inpatients admitted in different wards (Table 1).

**Table 1:** Clinical and demographic characteristics of the isolates

	*n* = 80	%
**Sample source**		
Pus	47	**58.8**
Urine	17	21.3
Blood	12	15.0
HVS	2	2.5
Stool	2	2.5
**Sex**		
Male	48	60.0
Female	32	40.0
**Age groups**		
Adult (≥ 18-year-old)	123	**87.9**
Children (< 18-year-old)	17	12.1
**Departments**		
Surgical	30	**37.5**
Medical ward	25	31.3
OPD	12	15.0
Gynecology	8	10.1
Pediatric	4	5.0
Orthopedic	1	1.3
**Type of patients**		
Inpatient	51	**63.8**
Outpatient	29	36.3
**Underlying medical conditions**		
Septic wound infection	39	**48.7**
Diabetic wound infection	8	10.0
Genitourinary infection	17	21.2
Septicemia	14	17.5
Gastritis	2	2.5

HVS: High Vaginal Swab, OPD: Outpatient Department

### Detection of virulent PVL encoding gene among the MRSA isolates

The majority 69 (86%) of the MRSA isolates in this study were not found to harbor the pvl (*LukS-PV* and *LukF-PV*) encoding genes in their genome while the above *pvl* encoding genes were only genetically found to reside in 11 (14%) of the MRSA isolates. Notably, among the isolates carrying the pvl-encoding genes for the production of potent pvl toxin, a substantial proportion of 54.5% were obtained from patients with wound infections.

### Distribution of antiseptic genes among the MRSA isolates

Of the 80 MRSA isolates, the majority 65 (81.3%) demonstrated genetic resistance to antiseptics/disinfectants. Specifically, these isolates demonstrated resistance to ethidium bromide, chlorhexidine, benzylkonium chloride, and cetylpyridinium chloride; commonly used disinfectant agents. Notably, this resistance was consistently associated with the presence of *qacD* genes across all isolates which demonstrated resistance to antiseptics. Additionally, among the pvl-positive MRSA isolates, a significant proportion (81.8%) harbored *qacD* genes.

### Genetic characteristics of sequenced MRSA isolates

**Fig. 1:**
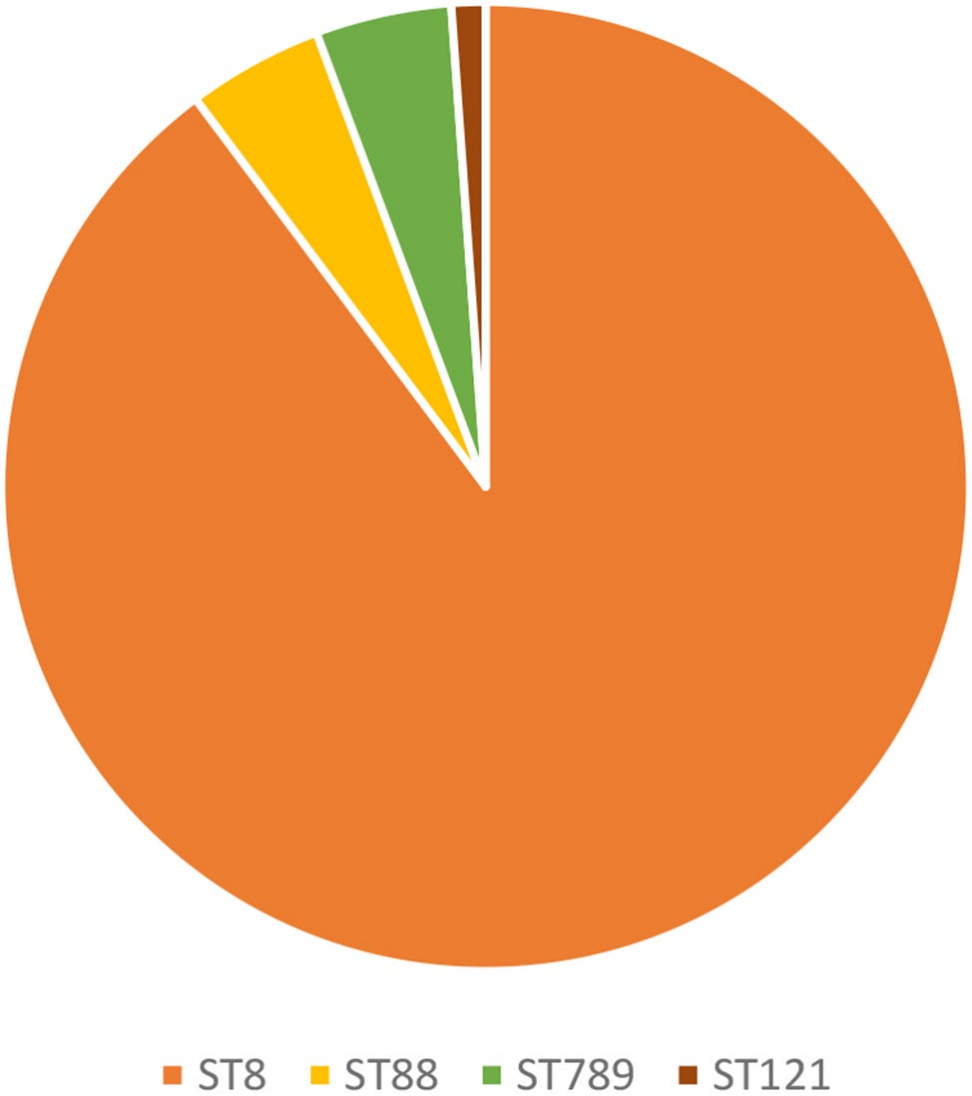
Distribution of Sequence types among the isolates

Multilocus Sequence Typing revealed that, majority of the isolates, 72 (90%) were identified as ST8, while ST789, ST88 and ST121 were 4 (5%), 3 (3.8%), and 1 (1.2%) respectively (Fig. 1). The majority 7 (63.6%) of pvl-carrying MRSA were identified as ST8 isolates. Nevertheless, SCC*mec* typing consistently identified SCC*mec* elements of type IV (subtype 2B&5) across all MRSA isolates.

### Virulence genes and resistance genes of the sequenced isolates

Genetically, the 11 pvl-carrying Methicillin-resistant *Staphylococcus aureus* isolates were found to harbor multiple genes including virulence and antibiotic resistance genes. The detected resistance genes were found randomly occurring among the isolates with variable genes combinations (Table 2).

**Table 2:** Multilocus sequence typing, virulence genes, antimicrobial and antiseptic resistance genes of the isolates with their hospitals of origins

Sample IDs	Hospital of origin	Species	MLST	Resistance genes	Virulence factors	Antiseptic gene
FFDD_125	Dodoma Regional Referral Hospital	*S. aureus*	ST-789	*aac (6’)-aph (2’’)*,* aph (3’)-III*,* blaZ*,* dfrG*,* erm(C)*,* mecA*,* mph(E)*,* msr (E)*,* tet(K)*	*aur*,* edinB*,* hlgA*,* hlgB*,* hlgC*,* lukD*,* lukE*,* lukF-PV*,* lukS-PV*,* sak*,* scn*,* sep*,* splA*,* splB*,* splE*	*qacD*
FFDD_2385	Dodoma Regional Referral Hospital	*S. aureus*	ST-8	*blaZ*,* mecA*	*aur*,* hlgA*,* hlgB*,* hlgC*,* lukD*,* lukE*,* lukF-PV*,* lukS-PV*,* sak*,* scn*,* splA*,* splB*	Not Found
FFMG_214-1-21	Morogoro Regional Referral Hospital	*S. aureus*	ST-8	*aac (6’)-aph (2’’)*,* blaZ*,* dfrG*,* erm(C)*,* mecA*	*aur*,* hlgA*,* hlgB*,* hlgC*,* lukD*,* lukE*,* lukF-PV*,* lukS-PV*,* sak*,* scn*,* sec*,* sej*,* sel*,* ser*,* splA*,* splB*,* splE*,* tst*	*qacD*
FFMG_347-07-21	Morogoro Regional Referral Hospital	*S. aureus*	ST-8	*aac (6’)-aph (2’’)*,* blaZ*,* dfrG*,* erm(C)*,* mecA*,* tet(K)*	*aur*,* hlgA*,* hlgB*,* hlgC*,* lukD*,* lukE*,* lukF-PV*,* lukS-PV*,* sak*,* scn*,* sej*,* ser*,* splA*,* splB*	*qacD*
FFMG_267-2-21	Morogoro Regional Referral Hospital	*S. aureus*	ST-8	*aac (6’)-aph (2’’)*,* blaZ*,* dfrG*,* erm(C)*,* mecA*	*aur*,* hlgA*,* hlgB*,* hlgC*,* lukD*,* lukE*,* lukF-PV*,* lukS-PV*,* sak*,* scn*,* sej*,* ser*,* splA*,* splB*,* splE*	Not Found
FFMG_55-7-21B-S-CO	Morogoro Regional Referral Hospital	*S. aureus*	ST-8	*aac (6’)-aph (2’’)*,* blaZ*,* dfrG*,* erm(C)*,* mecA*,* sul2*,* tet(K)*	*ElrA*,* SrtA*,* ace*,* astA*,* aur*,* cCF10*,* cad*,* camE*,* efaAfs*,* hlgA*,* hlgB*,* hlgC*,* lukD*,* lukE*,* lukF-PV*,* lukS-PV*,* sak*,* scn*,* sej*,* ser*,* splA*,* splB*,* tpx*	*qacD*
FFZB_150	Mnazi Mmoja Referral Hospital	*S. aureus*	ST-121	*aac (6’)-aph (2’’)*,* blaZ*,* dfrG*,* mecA*,* tet(K)*	*aur*,* hlgA*,* hlgB*,* hlgC*,* lukD*,* lukE*,* lukF-PV*,* lukS-PV*,* sak*,* scn*,* seb*,* seg*,* sei*,* sej*,* sem*,* sen*,* seo*,* sep*,* ser*,* seu*,* splA*,* splB*,* splE*	*qacD*
FFZB_192A-R57	Mnazi Mmoja Referral Hospital	*S. aureus*	ST-8	*blaZ*,* dfrG*,* mecA*,* tet(K)*	*aur*,* hlgA*,* hlgB*,* hlgC*,* lukD*,* lukE*,* lukF-PV*,* lukS-PV*,* sak*,* scn*,* sej*,* sep*,* ser*,* splA*,* splB*,* splE*	*qacD*
FFZB_200-C	Mnazi Mmoja Referral Hospital	*S. aureus*	ST-88	*aac (6’)-aph (2’’)*,* blaZ*,* dfrA17*,* dfrG*,* mecA*,* tet(K)*	*aur*,* hlgA*,* hlgB*,* hlgC*,* lukD*,* lukE*,* lukF-PV*,* lukS-PV*,* papA_F43*,* sak*,* scn*,* sej*,* sep*,* ser*,* shiB*,* splA*,* splB*,* splE*,* traT*	*qacD*
FFZB_64	Mnazi Mmoja Referral Hospital	*S. aureus*	ST-8	*aac (6’)-aph (2’’)*,* blaZ*,* dfrG*,* mecA*,* tet(K)*	*aur*,* hlgA*,* hlgB*,* hlgC*,* lukD*,* lukE*,* lukF-PV*,* lukS-PV*,* sak*,* scn*,* sej*,* sep*,* ser*,* splA*,* splB*,* splE*	*qacD*
FFZB_71	Mnazi Mmoja Referral Hospital	*S. aureus*	ST-88	*aac (6’)-aph (2’’)*,* blaZ*,* dfrG*,* mecA*,* tet(K)*	*aur*,* hlgA*,* hlgB*,* hlgC*,* lukD*,* lukE*,* lukF-PV*,* lukS-PV*,* sak*,* scn*,* sej*,* sep*,* ser*,* splA*,* splB*	*qacD*

### Phylogenetic relatedness among the *pvl*-positive MRSA isolates

The phylogenetic relationships among the 11 MRSA isolates carrying pvl-encoding genes within their genome were inferred. The genomes of these isolates were compared to a reference genome of a well-characterized ST8 USA300 (FPR3757) strain with accession number CP000255. The reference organism had a chromosomal length of 2,358,411 base pairs (bps). Collectively, all isolates covered 87.8% of the reference genome. Notably, a total of 2,072,683 positions were shared across all analyzed genomes. The SNPs analysis from this study detected the SNPs difference of 3 to 16,476.

**Fig. 2:**
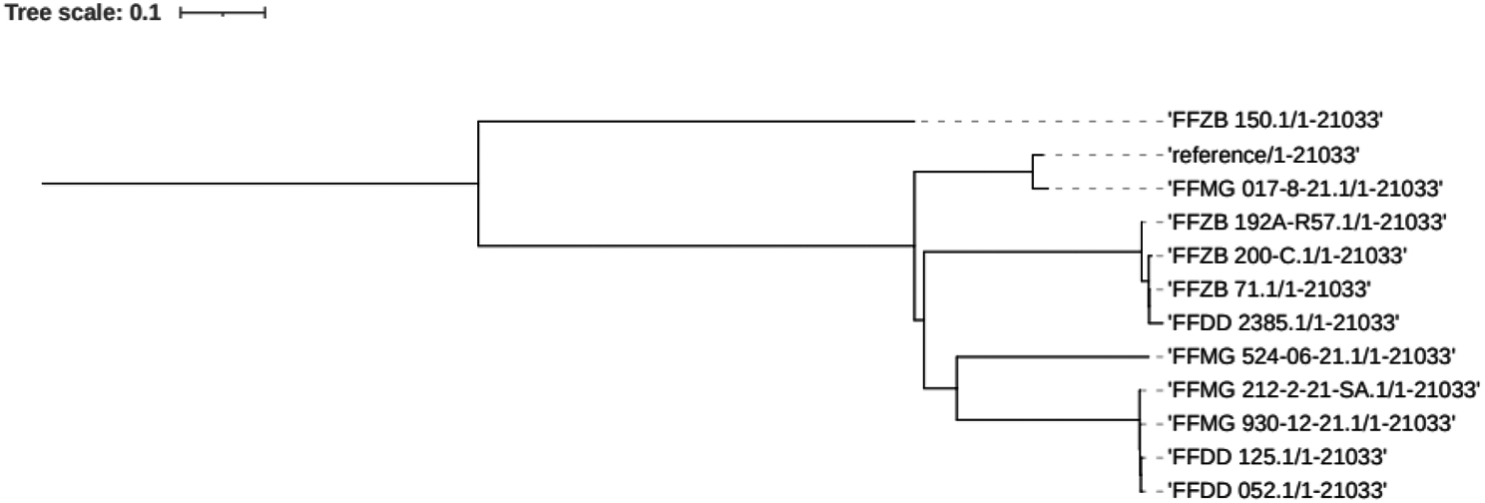
Phylogenetic tree of Pvl positive MRSA isolates

Two distinct clades were detected, one made up of four organisms while the other made up of two organisms. However, in their entirety, pvl-positive MRSA isolates from Morogoro formed one clade with minor pairwise SNP differences ranging from 3 to 82. The subsequent clade was found among the isolates from Mnazi Mmoja Hospital of Zanzibar, with SNP analysis demonstrating an SNP difference of 4. High SNPs difference ranging from 7577 to 8455 was detected among the two pvl-positive MRSA isolates from Dodoma Regional Referral Hospital. Regarding inter-hospital differences, SNP analysis revealed that the isolates from each hospital were genetically diverse from each other, evidently from their pairwise SNPs differences of 79 to 82. However, an exception was observed, whereas a single isolate from Zanzibar (FFZB_64.1) showed high genetic closeness with all four pvl-positive MRSA isolates from Morogoro hospital (Fig. 2).

## Discussion

The global spread of MRSA presents a serious public health challenge and a significant issue for healthcare providers. Various factors contribute to the drug resistance and pathogenicity of *S. aureus*. PVL-positive MRSA were first identified in the late 1990s, since then these strains have become widespread globally [26]. This study provides crucial insights into the genomic characteristics, virulence genes particularly PVL and antiseptic resistance profiles of methicillin-resistant *S. aureus* isolates from various regional referral hospitals in Tanzania. A total of 80 Methicillin-resistant *Staphylococcus aureus* isolates were confirmed using both phenotypic and genotypic methods, with the majority being sourced from wound pus, reflecting the high incidence of septic wound infections among the patients.

Our findings regarding the presence of Panton-Valentine genes are particularly noteworthy. The detection of *LukS-PV* and *LukF-PV* genes in 14% of the MRSA isolates aligns with global reports that link these genes to increased virulence and transmissibility [15,27). The majority of the pvl-carrying MRSA isolates were notably associated with wound infections, which is consistent with the role of Panton-Valentine Leukocidin in causing severe skin and soft tissue infections. Remarkably, this pore-forming protein, which is usually found in ST8 MRSA isolates, has also been found to exist in non-ST8 MRSA isolates in this study. The mere presence of *pvl* encoding genes in the genome of the already multidrug-resistant MRSA strains further amplifies their invasive properties and renders these bacteria nothing short of dangerous [27–29]. The potential effect of the Panton valentine leucocidins production owing to the presence of the pvl-encoding genes in MRSA is not only widening the virulence factors of these bacteria but has an incredible effect on disease severity, which in turn prolongs hospital stays. The fact that the majority of MRSA strains are readily resistant to many antibiotics [12, 30], pvl is thought to enhance the pathogenicity of *S. aureus* by causing tissue necrosis, accelerating cell apoptosis, and destroying white blood cells [28, 31]. This finding highlights the clinical significance of *LukS-PV* and *LukF-PV* genes in the pathogenesis of MRSA infections and underscores the need for targeted interventions to manage and control infection caused by these hypervirulent strains. The result is in contrast to another study done in Moshi Tanzania, which reported the majority of pvl-carrying bacteria being Methicillin Susceptible *S. aureus* [32]. This discrepancy can be explained by the potential *mecA* gene transmission ability, and high evolutionary and adaptability of methicillin-resistant *S. aureus* bacteria. The predominance of isolates from surgical wards (37.5%) aligns with the high rate of wound infections, underscoring the need for stringent infection control practices in these settings.

The presence of PVL among male patients was found to be slightly higher (60%) compared to female patients (40%). Although adults showed a higher prevalence of PVL compared to children, this difference was not statistically significant. This trend is consistent with findings from another study conducted in India [33]. Conversely, some studies have noted a stronger association of PVL with younger children. Among hospital units, the highest number of PVL-positive MRSA isolates (30/80) were collected from the surgical departments, followed by medical wards (25/80). A similar distribution pattern of PVL-positive MRSA isolates across hospital units has been reported in India [29]. It is worth considering that, the observed Panton Valentine Leucocidins encoding genes (*LukS-PV* and *LukF-PV*) are typically mostly found in Methicillin Susceptible *S. aureus* strains bacteria, less virulent strains readily susceptible to wide arrays of antibiotics [14, 18, 32, 34]. However, in this study *LukS-PV* and *LukF-PV* virulent genes were detected in Methicillin-resistant *S. aureus* strains (MRSA), which are known for their multidrug resistance and heightened virulence [24, 35]. The mere detection of this potential virulent pvl- encoding genes in however small number of isolates 11 (14%), underscores the growing concerns of MRSA strains acquiring additional virulence factors, thereby exacerbating their pathogenic potential [28]. Furthermore, the fact that bacteria can disseminate their genes through horizontal transmission [36], suggests a worrisome prospect of *LukS-PV* and *LukF-PV* genes dissemination among the MRSA bacterial strains. Therefore, proactive measures are imperative to contain this potential emerging threat and prevent its potential escalation.

The study also sheds light on the resistance to antiseptics and disinfectants among the isolates. A significant majority (81.8%) demonstrated resistance to antiseptics. The resistance was predominantly mediated by the *qacD* gene, which encodes for efflux pumps that reduce the efficacy of commonly used disinfectants such as ethidium bromide, chlorhexidine, benzalkonium chloride, and cetylpyridinium chloride. This high rate of antiseptic resistance is of concern, since it poses a significant challenge to infection control measures, as it limits the effectiveness of commonly used disinfectants that are crucial in preventing nosocomial infections [37]. The co-occurrence of *qacD* and pvl genes in 81.8% of already multidrug-resistant pvl-positive MRSA isolates further complicates treatment and control efforts, as these strains are both highly virulent and resistant to antiseptics and antibiotics [12, 38–40]. The resistance profile of these strains against several antibiotics used in clinical settings is alone growing at a worrying rate. However, the detection of antiseptic *qacD* genes within the genome of bacteria as demonstrated in this study can exacerbate the transmissibility of these bacteria among the patients. In this regard, resistance to common disinfectants/antiseptics will not only impose the potential spreading burden in our hospitals but further compromise our ability to control and treat infections caused by these strains. Moreover, this shed more light on the importance of strong antimicrobial stewardship and proper infection control practices in our hospitals. These findings significantly diverge from those reported in previous studies [41, 42] which reported *qacA*/*B* being the highly dominant antiseptic genes among the *S. aureus* isolates. The difference can be explained by differences in geographical location, sample size, and methods of detection used. The high prevalence of antiseptic resistance genes and the presence of virulence encoding genes among the pvl-positive MRSA isolates further emphasize the pressing need for enhancing antimicrobial stewardship and adequate infection control practices in healthcare settings. The high prevalence of antiseptic resistance genes and the presence of virulence encoding genes among the pvl-positive MRSA isolates underscores the pressing need for enhancing antimicrobial stewardship and adequate infection control practices in healthcare settings.

Genetic analysis through Multilocus Sequence Typing (MLST) identified that ST8 was the most predominant sequence type (90.1%), with other types such as ST789, ST88, and ST21 appearing less frequently. The dominance of ST8, particularly among PVL-positive MRSA isolates is consistent with the previous studies that have identified this sequence type as a common lineage associated with community-acquired Methicillin-Resistant *S. aureus* (CA-MRSA) infections [32, 43]. The presence of SCC*mec* type IV subtype 2B&5 and PVL in all isolates further supports their community origins and classification as CA-MRSA [31]. The phylogenetic analysis provided insights into the genetic diversity among the isolates. The genetic diversity observed among the pvl-carrying MRSA isolates, as evidenced by SNP analysis, indicates multiple sources, and evolutionary origins. This suggests the potential for ongoing transmission and genetic recombination within the hospital’s environment. The formation of distinct clades among the isolates from different hospitals, coupled with significant SNP differences, underscores the genetic variability and adaptability of MRSA. This genetic diversity, especially the close genetic relationship between isolates from Morogoro and Zanzibar, suggests regional dissemination patterns that may be influenced by patients’ movement and hospital referral practices.

The findings of this study have highlighted the significant implications for public health and hospital infection control practices in Tanzania. The high prevalence of antiseptic resistance among the MRSA isolates calls for a re-evaluation of current disinfection protocols and the development of alternative strategies to mitigate the spread of resistant strains. Enhanced surveillance and molecular characterization of MRSA isolates are essential in monitoring the emergence and dissemination of virulent and antiseptic-resistant strains. Furthermore, the detection of multiple virulence factors and resistance genes in PVL-positive MRSA isolates underscores the need for comprehensive infection control measures, including the judicious use of antibiotics and disinfectants, to prevent the selection and spread of these highly pathogenic strains.

### Conclusion and recommendations

The escalating rise of antiseptic resistance in bacteria is a pressing concern to public health. The co-occurrence of *pvl* and antiseptic resistance genes in a significant proportion of the MRSA isolates underscores the need for stringent infection control practices and robust antimicrobial stewardship programs to combat the threat posed by these formidable pathogens. Future studies should focus on exploring the mechanisms underlying antiseptic resistance and developing novel therapeutic strategies to effectively manage infections caused by *pvl*-positive MRSA strains. Comprehensive genomic studies which include whole genome sequencing and comparative genomic studies should also be done to detail, analyze, and characterize novel resistance determinants and virulence factors. Regarding resistance, strengthening antimicrobial stewardship programs to promote the proper use of antibiotics and disinfectants, reduces selective pressure for resistant strains. Lastly, providing comprehensive training for healthcare workers on infection control practices, including proper disinfection protocols and measures to prevent the spread of MRSA within healthcare facilities.

## Data Availability

The genome assemblies and raw reads of all eighty isolates from this study have been deposited in the European Nucleotide Archive (http://www.ebi.ac.uk/ena) under accession number PRJEB71932.
